# Pharmaceutical concentration using organic solvent forward osmosis for solvent recovery

**DOI:** 10.1038/s41467-018-03612-2

**Published:** 2018-04-12

**Authors:** Yue Cui, Tai-Shung Chung

**Affiliations:** Department of Chemical and Biomolecular Engineering, National University of Singapore, Singapore, 117585 Singapore

## Abstract

The organic solvent forward osmosis (OSFO) process can simultaneously concentrate the active pharmaceutical ingredients (APIs) and recover the organic solvents. Here we demonstrate and evaluate an OSFO process for solvent recovery. In this demonstration, OSFO was conducted in different solvents with different draw solutes. The OSFO process shows rejections >98% when recovering organic solvents from different feed solutions, even when the feed concentration is as high as 20 wt%. More importantly, all systems exhibit relatively low ratios of reverse solute flux to solvent flux, indicating that the adverse effects of using hazardous draw solutions could be minimized. Nevertheless, the use of non-hazardous draw solutes such as citric acid is highly recommended to remove any potential risk, and it has been demonstrated. Herein, the OSFO process is a promising technology for solvent recovery as it possesses a reasonable solvent flux, low reverse solute flux and requires no external pressure.

## Abstract (web summary)

Solvent recovery is an important process in the pharmaceutical industry, but organic solvent nanofiltration membranes operate under high pressures. Here the authors demonstrate organic solvent forward osmosis — an alternative process that does not require application of external pressure and may prove to be economically favorable.

## Introduction

The syntheses of active pharmaceutical ingredients (APIs) usually requires multi-step molecular constructions of target compounds in different organic solvents^1–5^. Thus, multiple-stage purification of pharmaceuticals and their intermediates are necessary, which makes the removal of organic solvents an essential step in pharmaceutical syntheses. The majority of waste solvents are usually sent to on-site incineration due to the high cost of solvent recovery via conventional methods, such as distillation. However, with stricter environmental legislation and increasing prices of organic solvents, solvent recovery is vital and becomes a competitive alternative to incineration^6^. Additionally, most pharmaceutical products are highly temperature sensitive, and they may decompose or de-nature under high temperatures^4, 7, 8^. As a result, single-step athermal separation processes to simultaneously recover the solvents and concentrate the pharmaceutical products are attractive.

Membrane technologies have a significant growth in past decades due to their unique characteristics, such as small footprint, lower energy consumption and no phase transformation involved during separation^9, 10^. Among various membrane technologies, organic solvent nanofiltration (OSN) is commonly used for organic solvent recovery and pharmaceutical concentration^7, 11, 12^. However, the high pressure used in OSN processes remains a concern because it may add extra operating and maintenance costs. Thus, a more cost viable and sustainable technology is needed to concentrate the pharmaceutical products while recovering the solvents.

Forward osmosis (FO) has received great attention in the last decade for water reuse and seawater desalination^13–17^. Unlike conventional pressure-driven membrane processes such as reverse osmosis (RO) and nanofiltration (NF) which utilize external hydraulic pressure, FO takes the advantage of chemical gradient across a semi-permeable membrane to transport water from the low concentration side to the high concentration side^18–20^. Since FO does not employ external pressure, it offers a number of advantages over the pressure driven processes. For example, FO exhibits lower fouling tendency, compared to the direct filtration process, for the same aqueous feed solution. Furthermore, the flux recovery of FO is comparatively higher than the direct filtration process^[[15,21–24^. This may potentially lower the operational and maintenance costs. Valladares Linares et al.^24^ have done a detailed economic analysis on capital and operational expenses (CAPEX and OPEX) for a hybrid FO–low-pressure RO (FO-LPRO) process and a conventional seawater reverse osmosis (SWRO) desalination process. They found that the FO-LPRO systems have a 21% higher CAPEX, a 56% lower OPEX and a total cost reduction of 16% compared to SWRO due to the savings in energy consumption and fouling control.

Utilizing the same principle, organic solvent forward osmosis (OSFO), which has been proposed and first described in details by Lively and Sholl^25^, can concurrently concentrate the pharmaceutical products in the feed side while transporting the organic solvent to the draw solution. Subsequently, the diluted draw solution can be regenerated by many means, such as direct filtration, distillation and evaporation^26–28^. Since no external pressure is applied, the use of OSFO may potentially reduce the fouling tendency and/or the irreversible fouling, which may lower the operating cost when comparing with OSN. In addition, OSFO can be utilized in challenging separations where the osmotic pressure of the mother liquor is prohibitively high for pressure driven processes^25^. However, it should be noted that although the basic principles are similar, there are differences between the applications of FO in water and in organic solvents. Thus, many aspects, such as energy consumption and/or cost comparison between OSFO and conventional methods, draw solution regeneration, fouling, and hybrid systems, should be further studied.

Nevertheless, the objective of this work is to first demonstrate the feasibility of using OSFO for organic solvent recovery. A thin film composite (TFC) membrane would be employed as the semi-permeable membrane. It consists of a polyamide selective layer and a porous polyimide substrate. To ensure its chemical stability in various organic solvents, the substrate would be first crosslinked with diamine. A polyamide selective layer would then be formed on top of the crosslinked substrate via interfacial polymerization^29–31^. This type of membranes has been proven to be relatively stable in a variety of solvents^32^. Subsequently, solvent recovery via OSFO has been demonstrated using different kinds of draw solutions in various solvent systems. This work may open up a totally new research area for the recovery of organic solvents via OSFO for the pharmaceutical industry.

## Results

### Solvent recovery

**Fig. 1:**
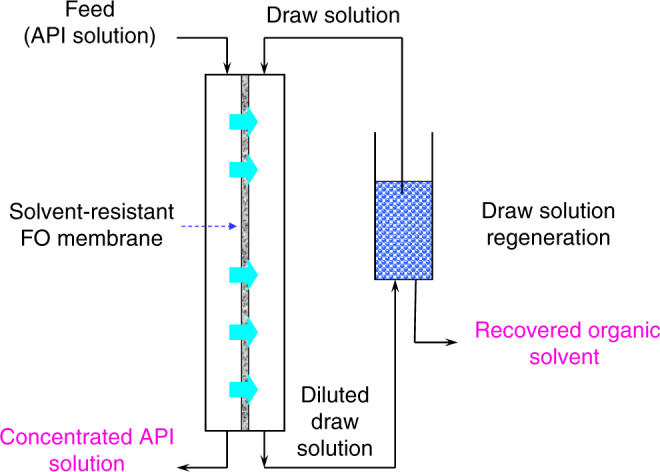
Schematic diagram of a forward osmosis process for organic solvent recovery. At the membrane compartment, the solvent from the feed solution transports through the membrane to the draw solution while the solutes are rejected. As a result, the feed solution is concentrated while the draw solution is diluted. Subsequently, the diluted draw solution can be regenerated by many means, such as direct filtration, distillation and evaporation

The use of OSFO processes for the simultaneous concentration of pharmaceutical products and recovery of organic solvents has been demonstrated in Fig. 1.

**Fig. 2:**
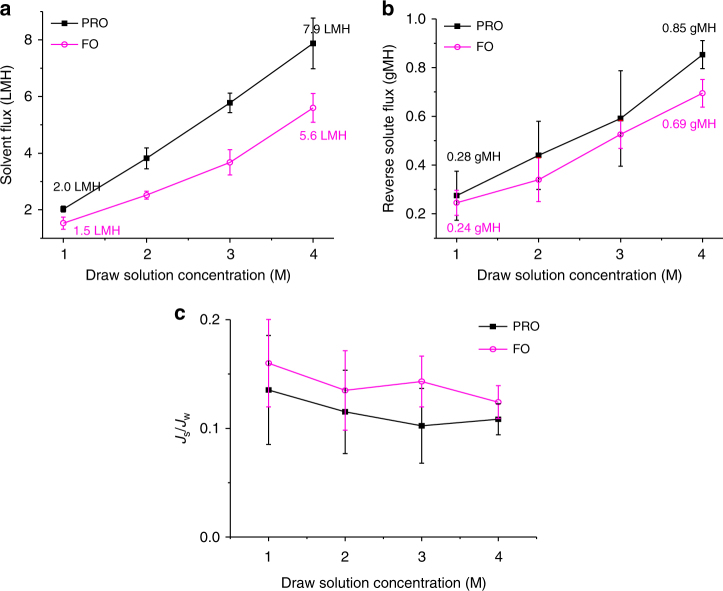
Assessment of the effect of the draw solution concentration. **a** Solvent flux, **b** reverse solute flux and **c** selectivity *J*_s_/*J*_w_ as a function of draw solution concentration where the feed solution is pure ethanol and the draw solution is LiCl in ethanol. The error bar represented the standard deviation from at least three independent OSFO tests with different membrane coupons, and the interfacial polymerization was conducted separately for each membrane coupon. Our results demonstrate that the solvent flux and the reverse solute flux increase proportionally to the draw solution concentration but the membrane selectivity is not compromised

The OSFO ability to recover solvents from pharmaceutical syntheses is first evaluated using pure ethanol as the model organic feed solution. Lithium chloride (LiCl) is utilized as the model draw solute in this study due to its ease of detection. As shown in Fig. 2a and Table 1, a positive ethanol flux is observed under this process, indicating that ethanol is indeed drawn from the feed side to the draw side. This observation is in accordance with the natural osmosis process where the solvent molecules spontaneously transport against the osmotic pressure gradient to equalize the solute concentrations on both the sides^33^. However, the ethanol flux is not impressively high, compared to aqueous systems. This is probably because the highly crosslinked substrate and selective layer not only limit the membrane swelling but also introduce a relatively high resistance for ethanol transport. In addition, as indicated in Supplementary Table 1, the relatively large size (i.e. 4.5 Å) and high viscosity (i.e., 1.10 mPa s) of ethanol, compared to those of water (i.e., 2.75 Å and 0.89 mPa s, respectively), might make difficulties for its molecules to permeate through the membrane. This hypothesis is supported by the relatively low ethanol permeance of 0.23 L m^-2^ h^-1^ bar^-1^ (LMH bar^−1^), as exhibited in Table 2. Hence, the ethanol transport across the membrane is slightly retarded and a relatively low ethanol flux is observed.

**Table 1:** Membrane performance in different solvent systems

Solvent	Draw solution	Osmotic pressure of draw solution (Bar)	Membrane orientation	Solvent flux (LMH)	Reverse solute flux (gMH)	*J*_s_/*J*_w_
Ethanol	2 M LiCl	50.2 ± 1.1	PRO	3.82 ± 0.37	0.44 ± 0.14	0.11 ± 0.03
			FO	2.52 ± 0.14	0.34 ± 0.09	0.13 ± 0.04
IPA	2 M LiCl	19.4 ± 4.6	PRO	0.51 ± 0.11	0.15 ± 0.09	0.28 ± 0.19
			FO	0.34 ± 0.06	0.11 ± 0.04	0.35 ± 0.13
Hexane	50 wt% Methyl Palmitate	8.8 ± 1.9	PRO	2.04 ± 0.34	N.D.	N.A.
			FO	1.87 ± 0.13	N.D.	N.A.

Feed solution: the respective pure solvent

**Table 2:** Solvent transport properties in the membrane

Solvent	Permeance (A, LMH bar^−1^)	Permeability (10^−9^, LMH bar^−1^ m)	Solubility (g g^−1^)	Diffusivity (10^−9^, m^2^ s^−1^)
Ethanol	0.230 ± 0.030	38.76 ± 6.78	18.76 ± 6.04	8.68 ± 2.97
Isopropanol	0.049 ± 0.013	8.19 ± 2.34	7.76 ± 3.61	3.33 ± 1.13
Hexane	0.027 ± 0.008	4.52 ± 1.46	1.92 ± 0.34	3.42 ± 1.29

For a comprehensive evaluation of OSFO for solvent recovery, the solvent flux (*J*_w_, L m^−2^ h^−1^, LMH), reverse solute flux (*J*_s_, g m^−2^ h^−1^, gMH) and membrane selectivity (*J*_s_*/J*_w_) are investigated as a function of LiCl concentration under both pressure-retarded osmosis (PRO) and FO modes. As exhibited in Fig. 2, an ethanol flux of 3.82 LMH and a reverse solute flux of 0.44 gMH are achieved under the PRO mode when 2 M LiCl in ethanol is employed as the draw solution. On the other hand, the ethanol flux and the reverse solute flux are slightly lower under the FO mode. They are only 2.52 LMH and 0.34 gMH, respectively, because of severe internal concentration polarization (ICP) as previously observed in water reuse and seawater desalination^34, 35^. As a result, the draw solution is severely diluted in the porous substrate under the FO mode and the effective osmotic pressure gradient across the membrane is harshly reduced. However, impressively low *J*_s_*/J*_w_ values of 0.11 and 0.13 are achieved under the PRO and FO modes, respectively, indicating the OSFO process has a considerably high selectivity of draw solute over solvent. In summary, ethanol can be extracted from pure ethanol systems with a reasonable flux and a minimal reverse flux of the draw solute by means of the OSFO process. Therefore, the OSFO process is a potential method for organic solvent recovery.

Figure 2 also shows the ethanol flux increases almost linearly from 2.0 to 7.9 LMH under the PRO mode, while it augments from 1.5 to 5.6 LMH under the FO mode when the LiCl concentration increases from 1 to 4 M. Their corresponding reverse solute fluxes exhibit a similar increasing trend. The former varies from 0.28–0.85 gMH, while the latter from 0.24 to 0.69 gMH. However, their *J*_s_/*J*_w_ ratios fluctuate within a relatively narrow range, indicating that the membrane has a high selectivity even when the draw solution concentration increases. In general, the ethanol flux under the PRO mode increases at a faster rate than that under the FO mode. This arises from the fact that the FO mode has more severe ICP effect than the PRO mode. In addition, ICP is further intensified when the draw solution becomes more concentrated. As a result, the FO mode has a lower effective concentration gradient (i.e., osmotic pressure gradient) across the membrane than the PRO mode, especially when using a highly concentrated draw solution. The corresponding reverse solute fluxes also display a similar trend. However, the membrane selectivity is not compromised and the *J*_s_/*J*_w_ values remain relatively constant. Therefore, one may be able to use a more concentrated draw solution to enhance the solvent recovery rate by the OSFO process.

Since the pharmaceutical industry utilizes a wide variety of organic solvents besides ethanol, it is necessary to evaluate the OSFO performance in different solvent systems. Thus, OSFO performance in both IPA and hexane systems is examined. In this section, only pure solvent is adopted as the feed solution.

Similar to the ethanol system, pure IPA and 2 M LiCl dissolved in IPA are used as the feed and draw solutions for the IPA system, respectively. Table 1 summarizes the experimental results. An IPA flux of 0.51 LMH under the PRO mode and a slightly lower flux of 0.34 LMH under the FO mode are obtained. Similarly, the flux difference is attributed to the ICP effect. The reverse solute fluxes are as low as 0.15 and 0.11 gMH under the PRO and FO modes, respectively. In the hexane system, owing to the limited solubility of LiCl in hexane, 50 wt% methyl palmitate is adopted as the draw solution. Pure hexane is utilized as the feed solution. The obtained hexane fluxes are 2.04 LMH under the PRO mode and 1.87 LMH under the FO mode. Besides, the reverse solute flux of methyl palmitate is negligible as its concentration in the feed solution is undetectable by the UV–vis spectrometer.

Clearly, OSFO is applicable to not only the ethanol system but also the IPA and hexane systems, and potentially for other solvent systems if appropriate draw solutes are available. However, the membrane performance varies significantly in different solvent systems. For instance, the ethanol flux is much higher than the IPA flux despite using the same draw solution concentration (i.e., 2 M LiCl). This is likely because of two reasons. First, the permeability of ethanol (38.76 × 10^−9^ L m^−2^ h^−1^ bar^−1^ m, LMH bar^−1^ m) is higher than that of IPA (8.19 × 10^−9^ LMH bar^−1^ m), as exhibited in Table 2. In addition, the osmotic pressures generated by the same molar amount of LiCl in the two systems are different. As shown in Table 1, the measured osmotic pressure of a 2 M LiCl ethanol solution is much higher than that of a 2 M LiCl IPA solution. Theoretically, the osmotic pressures of 2 M LiCl in both solvents should be identical according to the original van't Hoff’s equation *π* = *icRT*^33, 34^. However, LiCl may undergo a higher degree of dissociation and generate more ion species in ethanol than in IPA due to the higher polarity of ethanol^36^. This hypothesis is confirmed because 2 M LiCl in ethanol has a much higher conductivity (6.78 ± 0.03 ms cm^−1^) than that in IPA (1.18 ± 0.01 ms cm^−1^). Generally, the higher conductivity, the greater amount of ion species in the solution^37, 38^. As such, LiCl in ethanol generates a higher osmotic pressure than that in IPA. In addition, it is interesting to observe that the measured osmotic pressure is much lower than the theoretical value calculated based on the van’t Hoff’s equation. This difference may arise from the concentration polarization at the membrane interface during the measurements, the non-ideality of the draw solution owing to its high concentration and the leakage of the draw solutes to the solvent^39^.

However, the solvent permeability and osmotic pressure may not be the only factors determining the solvent flux. As illustrated in Table 1, the 50 wt% methyl palmitate/hexane draw solution actually possesses a much lower osmotic pressure than the 2 M LiCl/IPA solution (8.8 ± 1.9 bar vs. 19.4 ± 4.6 bar), but the hexane flux is significantly higher than the IPA flux (2.04 ± 0.34 LMH vs. 0.51 ± 0.11 LMH under the PRO mode). In order to gain a better understanding of the solvent transport mechanisms across the membrane, the solution-diffusion model is used to evaluate the permeability, solubility and diffusivity of the solvents across the membrane, as displayed in Table 2. Interestingly, IPA has a higher permeability (8.19 × 10^−9^ LMH bar^−1^ m) than hexane (4.52 × 10^−9^ LMH bar^−1^ m), which is different from the order of their fluxes. This highlights the limitations of using the solution diffusion model in organic solvent systems, and there is a need to develop a new rigorous model. This discrepancy may arise from the fact that hexane has a much lower viscosity than IPA, as shown in Supplementary Table 1, which enables hexane to permeate through the membrane more easily. In addition, a comparison of solvent flux between PRO and FO modes in Table 1 also implies that the hexane system has another advantage over the ethanol and IPA systems. The former has a smaller percentage difference in solvent flux between PRO and FO modes than the latter because of a lower ICP effect. The satisfactory results from the three systems indicate that one may employ OSFO to recover a number of solvents with enhanced separation performance.

### Concurrent solvent recovery and pharmaceutical concentration

**Fig. 3:**
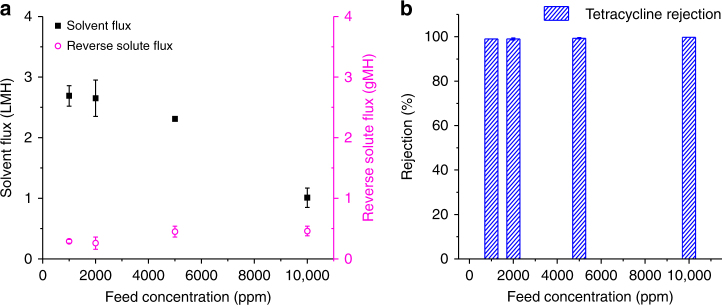
Assessment of the effect of the feed solution concentration. **a** Solvent flux and reverse solute flux as a function of tetracycline concentration. **b** Tetracycline rejections under different tetracycline concentrations. The draw solution is 2 M LiCl in ethanol and the operation mode is the FO mode. The error bar represented the standard deviation from at least three independent OSFO tests with different membrane coupons, and the interfacial polymerization was conducted separately for each membrane coupon. Our results indicate that the OSFO process is able to concentrate a pharmaceutical product up to 10000 ppm without sacrificing the rejection

To demonstrate OSFO capability to simultaneously concentrate pharmaceutical products and recover organic solvents, an API solution consisting of 2000 ppm tetracycline (*M*_w_ = 444.4 g mol^−1^) in ethanol and a 2 M LiCl ethanol solution were employed as the feed and draw solutions, respectively. As displayed in Fig. 3 and Table 3, the rejection of tetracycline is above 99%, while the ethanol flux and reverse solute flux under the FO mode are around 2.60 LMH and 0.26 gMH, respectively. The high rejection of tetracycline may arise from several factors. First, the highly crosslinked membrane structure offers significant resistance for tetracycline transport. Secondly, the dominant transport mechanism in OSFO is likely to be the solution diffusion mechanism^13, 40, 41^. To verify this hypothesis, the tetracycline rejection as a function of external pressure has been measured and summarized in Supplementary Fig. 1. The tetracycline rejection under the pressure driven process is around 80% at 1 bar and it is much lower than the rejection of 99% obtained under the OSFO process. More importantly, the rejection decreases drastically as the external pressure increases. This suggests that the pore flow mechanism plays a significant role in the pressure driven process while its influence is much weakened in the OSFO process^40, 42^. For comparison, the rejection of DuraMem 300 is also tested under the OSN process and listed in Supplementary Table 2. The lower rejection than the OSFO process further confirms our hypothesis. As such, the tetracycline transport across the membrane in the OSFO process would be mainly affected by its diffusivity and solubility. Tetracycline may have a reasonably low diffusivity because of its large solvated size in ethanol. Moreover, based on their Hansen solubility parameters calculated from the group contribution method, the solubility parameter difference between tetracycline and the membrane is relatively large (i.e., 28.7 vs. 23 MPa^1/2^)^43, 44^. Therefore, they have a low affinity towards each other and the tetracycline solubility in the membrane might be small^36, 45^. The combination of low diffusivity and solubility leads to a relatively low tetracycline flux of 0.098 LMH (as revealed in Supplementary Table 3 and the determination method can be found in Supplementary Methods) and thus, a high membrane rejection of tetracycline. In addition, since the reverse solute flux has an opposite transport direction to the solvent flow as well as the tetracycline molecules, it may also retard tetracycline transport through the membrane.

**Table 3:** Membrane rejection in different solvent systems

Solvent	Draw solution	Feed solution	Solvent flux (LMH)	Rejection (%)
Ethanol	2 M LiCl	2000 ppm Tetracycline	2.65 ± 0.30	99.0 ± 0.5
Isopropanol	2 M LiCl	2000 ppm Tetracycline	0.36 ± 0.15	99.2 ± 0.2
Hexane	50% Methyl palmitate	20% Triglycerides	1.35 ± 0.17	98.2 ± 0.6

To explore the extent to which the feed solution can be concentrated while still sustaining a reasonably high rejection of tetracycline, Fig. 3 investigates the ethanol flux and tetracycline rejection as a function of feed concentration using a draw solution concentration of 2 M LiCl. The ethanol flux gradually drops from 2.6 to 1.0 LMH when the feed concentration increases from 1000 to 10000 ppm owing to the loss of osmotic pressure gradient across the membrane. On the other hand, the rejection of tetracycline remains constantly higher than 99%. This impressive rejection indicates that the OSFO process is able to concentrate a pharmaceutical product up to 10000 ppm without sacrificing the rejection

For a comprehensive evaluation of OSFO capability to concentrate pharmaceutical products, solvent recovery from two other model solutions; namely, tetracycline in IPA and triglycerides in hexane, are also investigated. By using 2000 ppm tetracycline in IPA and 2 M LiCl/IPA solution as the feed and draw solutions, respectively, Table 3 shows a rejection of 99.2% is achieved with an IPA flux of 0.36 LMH. Since the mother liquor concentration may be as high as 20 wt% in real industrial applications, the hexane recovery is conducted using 20% triglycerides and 50% methyl palmitate as the feed and draw solutions, respectively. As revealed in Table 3, an impressively high rejection of 98.2% is obtained with a reasonable hexane flux of 1.35 LMH. This evinces the capability of OSFO to recover organic solvents from a concentrated mother liquor with a satisfactory rejection. The high rejection can be attributed to the highly crosslinked membrane structure, the unique FO transport mechanism and the hindrance from the reverse solute flux.

The satisfactory high rejections and reasonable solvent fluxes confirm that OSFO has real potential to recover organic solvents from pharmaceutical syntheses without much loss of the APIs.

### Alternative draw solutes in OSFO processes

Given that LiCl is found to be harmful if swallowed or inhaled, utilizing LiCl as the draw solute in OSFO processes may pose certain health hazards, regardless of the low reverse solute flux. Consequently, an additional step of removing LiCl from the pharmaceutical solution is required and it will incur extra cost. Thus, non-toxic compounds such as pharmaceutical excipients including diluents (e.g. sucrose), disintegrants (e.g. sodium starch glycolate) and binders (e.g. polyvinyl pyrrolidone) may be potential candidates as draw solutes^46^. Use of such compounds will eliminate any risks brought about by the reverse solute flux. Therefore, non-toxic pharmaceutical excipients, citric acid (CA), polyethylene glycol 1000 (PEG 1000) and diethanolamine (DEA), are evaluated as alternative draw solutes in this study. Table 4 summarizes their OSFO results. For the ethanol system using 2 M CA in ethanol as the draw solution, the ethanol fluxes of 2.85 and 2.05 LMH are attained under PRO and FO modes, respectively. In contrast, lower solvent fluxes of 1.23 and 0.89 LMH are obtained under PRO and FO modes, respectively, using 100 g L^−1^ PEG 1000 in ethanol as the draw solution. Compared to the previous LiCl case, the ethanol flux follows the sequence of 2 M LiCl > 2 M CA > 100 g L^−1^ PEG 1000. This order is exactly the same as the order of their corresponding osmotic pressures, as tabulated in Tables 1 and 4. Clearly, for the ethanol system, the ethanol flux is mainly determined by the osmotic pressure difference.

**Table 4:** Membrane performance with alternative draw solutions

Solvent	Alternative draw solution	Osmotic pressure of draw solution (Bar)	Membrane orientation	Solvent flux (LMH)	Reverse solute flux (gMH)	*J*_s_/*J*_w_
Ethanol	2 M CA	32.0 ± 4.6	PRO	2.85 ± 0.72	0.67 ± 0.02	0.24 ± 0.06
			FO	2.05 ± 0.30	0.53 ± 0.05	0.22 ± 0.04
	100 g L^-1^ PEG1000	5.4 ± 0.2	PRO	1.23 ± 0.19	0.90 ± 0.08	0.73 ± 0.13
			FO	0.89 ± 0.24	0.79 ± 0.09	0.88 ± 0.26
IPA	2 M DEA	4.2 ± 0.3	PRO	0.52 ± 0.05	N.D.	N.A.
			FO	0.32 ± 0.07	N.D.	N.A.

Feed solution: the respective pure solvent

Interestingly, the situation changes for the IPA system. As shown in Tables 1 and 4, the IPA fluxes are almost the same when using 2 M LiCl in IPA and 2 M DEA in IPA as draw solutions despite the former has a much higher osmotic pressure than the latter. One plausible explanation is due to the fact that IPA has a higher viscosity so that the solvent flux may be predominantly determined by the high solvent viscosity.

Since the reverse solute fluxes of these alternative draw solutions are considerably low, it guarantees the safety of utilizing OSFO to recover organic solvents in the pharmaceutical industry. Therefore, a variety of non-toxic compounds can be adopted as draw solutes for OSFO processes.

## Discussion

In this study, we have demonstrated the utilization of OSFO processes for organic solvent recovery from pharmaceutical products in various organic solvent systems including ethanol, IPA and hexane by using draw solutes, such as LiCl, CA and methyl palmitate. Unlike conventional pressure driven membrane separation processes, the OSFO process takes advantage of chemical potential difference across the membrane to facilitate solvent transport without any external hydraulic pressure, which may lower the operation and maintenance costs.

In this demonstration, ethanol fluxes of 3.82 and 2.52 LMH were attained under PRO and FO modes, respectively, using 2 M LiCl in ethanol as the draw solution and pure ethanol as the feed. In addition, the solvent flux is observed to increase proportionally to draw solution concentration. However, the effect of ICP on solvent flux was observed when using a highly concentrated draw solution. Additionally, positive results are also achieved in IPA and hexane systems, indicating that this process is applicable to a wide range of solvents. Generally, the solvent permeability and the osmotic pressure gradient across the membrane are the two major factors determining the solvent flux across a membrane. However, the solvent properties, such as viscosity, may also play a significantly role on solvent flux. In addition, solvent recovery from model solutions have been demonstrated. In the tetracycline/ethanol and tetracycline/IPA systems, tetracycline rejections of above 99% were obtained with reasonable solvent fluxes. In the triglycerides/hexane system, a rejection of triglycerides higher than 98% could be acquired even when a concentrated feed solution of 20% was utilized. These results suggest that OSFO has capability to concurrently concentrate pharmaceutical products and recover organic solvents. Furthermore, all studied systems showed relatively low ratios of reverse solute flux to solvent flux. This characteristic is highly important because it can minimize the potential hazards from the reverse solute flux to the feed solution. Alternative draw solutes to LiCl have also been examined in both ethanol and IPA. Encouraging results imply that a number of non-toxic compounds can be potentially employed as the draw solutes in the OSFO process to further eliminate the potential health hazards posed by the draw solute leakage. In summary, the reasonable solvent flux, low reverse draw solute flux and no applied pressure may bring the OSFO process as the next-generation technology for solvent recovery in the pharmaceutical industry.

However, despite the advantages of the OSFO process, such as low fouling tendency, minimal irreversible fouling and capability to treat highly concentrated feed solutions, the OSFO process suffers from certain drawbacks. For example, the solvent flux needs to be further improved. Nevertheless, this is only a starting point on OSFO for organic solvent recovery, future works should focus on development of high-performance membranes, studies of transport and separation mechanisms, discovery of other non-toxic compounds as draw solutes, system integration with draw solution regeneration and mathematical simulation of the integrated system to enhance the efficiency of solvent recovery, pharmaceutical concentration and draw solution regeneration.

## Methods

### Materials

The polyimide polymer Matrimid^®^ 5218 (Vantico Inc.), solvent N-methyl-2-pyrrolidinone (NMP, >99.5%, Merck) and non-solvent polyethylene glycol 400 (PEG 400, *M*_w_ = 400 g mol^−1^, Merck) were utilized to fabricate the membrane substrate. 1, 6-Hexanediamine (HDA, >98%) was purchased from Alfa-Aesar to crosslink the substrate. M-phenylenediamine (MPD, >99%), trimesoylchloride (TMC, >98%) and sodium dodecyl sulfate (SDS, >99%) were ordered from Sigma-Aldrich and employed for the interfacial polymerization reaction. LiCl (>99%, Sigma-Aldrich), CA (>99%, Sigma-Aldrich), DEA (>99%, Sigma-Aldrich), PEG 1000 (*M*_w_ = 1000 g mol^−1^, Merck) and methyl palmitate (>97.0%, Tokyo Chemical Industry) were utilized as the draw solutes. Ethanol (HPLC grade), isopropanol (IPA, HPLC grade), and n-hexane (HPLC grade) were ordered from Fisher Scientific and employed as the solvents to evaluate the membrane performance. Tetracycline (≥98.0 %, Sigma-Aldrich) and industrial sample, triglycerides from soybean oil (liquid, GIIAVA Singapore), were used as the model feed solutes. The commercial OSN membrane DuraMem 300 obtained from Evonik was utilized for transport mechanism studies. The deionized (DI) water was produced by a Milli-Q ultrapure water system (Millipore, USA). All chemicals were used as received.

### Fabrication of the crosslinked Matrimid substrate

The flat sheet membranes were prepared by a solution casting process, followed by the non-solvent induced phase inversion^43^. First, the Matrimid^®^ 5218 polymer was dried overnight at 80 °C in a vacuum oven to remove moisture and then dissolved in NMP with PEG 400 at 70 °C overnight at a weight ratio of 18/16/66 Matrimid^®^/PEG 400/NMP. Subsequently, the polymer solution was cooled down to room temperature and degassed for overnight before being cast by a casting knife onto a glass plate. The nascent membrane was immersed in DI water to form an asymmetric structure and then soaked in DI water to remove the residual NMP and PEG 400. Subsequently, the as-cast flat sheet membrane was crosslinked in a 5% HDA water/IPA (50:50) bath for 24 h, followed by thoroughly rinse in pure ethanol and preserved in DI water.

### Interfacial polymerization of TFC membranes

The formation of a thin polyamide layer on top of crosslinked Matrimid substrates was achieved via interfacial polymerization between MPD in an aqueous phase and TMC in an organic phase. The crosslinked Matrimid substrate was first immersed in a 2% MPD aqueous solution (containing 0.1% SDS) for 120 s. The excess MPD solution on membrane surface was removed with filter papers. A 0.1% TMC hexane solution was then deposited on top of the MPD-saturated substrate for 60 s. Subsequently, the membrane was air-dried for 5 min to complete the interfacial polymerization. The resultant TFC membrane was rinsed with ethanol to remove any residue and then preserved in a respective organic solvent (i.e., ethanol, IPA or hexane) which it would be tested subsequently.

### Solvent reclamation through OSFO

The organic solvent was reclaimed via OSFO using a lab-scale OSFO unit similar to a typical FO unit for water reuse^40, 42^ except that the system components are solvent resistant. The volumetric flows of both draw and feed solutions were kept at 0.2 L min^−1^. They were circulated in the setup using a pump and flowed countercurrently through the OSFO cell. Similar to the FO process for water reclamation, both the PRO mode (i.e., the selective layer faces the draw solution) and the FO mode (i.e., the selective layer faces the feed solution) were adopted. The whole system was stabilized for 0.5 h before measurements. Subsequently, the solvent flux (*J*_w_, L m^−2^ h^−1^, LMH) and reverse solute flux (*J*_s_, g m^−2^ h^−1^, gMH) were determined. The solvent flux was calculated from Eq. (1):

1 $$J_{\mathrm w} = \frac{{\Delta m}}{{{\mathrm{\rho }}\Delta t}}\frac{1}{{A_{\mathrm m}}},$$

where *A*_m_ is the effective cell area of 4 cm^2^; Δ*m* (g) is the average of the absolute weight loss in the feed side and the absolute weight gain in the draw side, *ρ* (g cm^−3^) is the solvent density, and Δ*t* (h) is the test duration of 2 h. After each test, a certain amount of the draw solute was added into the draw solution to maintain its concentration.

The reverse solute flux (*J*_s_) of the draw solution is calculated from the concentration increment in the feed solution using Eq. (2):

2 $$J_{\mathrm s} = \frac{{\Delta C_{\mathrm t}V}}{{\Delta t}}\frac{1}{{A_{\mathrm m}}},$$

where Δ*C*_t_ (g L^−1^) and *V*(L) are the changes of solute concentration and feed solution volume, respectively.

In OSFO tests, several compounds, such as LiCl, CA, DEA, PEG 1000 and methyl palmitate, were explored as draw solutes. However, their concentrations were not the same, as different compounds had different solubility in different solvents. Consequently, the concentrations of these compounds in the feed solutions after the tests were determined via different analytic methods: A conductivity meter (Metrohm, Switzerland) was utilized to determine the LiCl concentration. The CA concentration was measured by a high-performance liquid chromatography (HPLC, Agilent, USA) coupled with a variable wavelength detector (VWD) at the wavelength of 212 nm. On the other hand, the DEA concentration was determined by a gas chromatography (GC, Agilent) coupled with a flame ion detector (FID). The PEG 1000 concentration was measured with the aid of a total organic carbon analyzer (TOC, ASI-5000A, Shimazu, Japan). The feed solution was first rotary evaporated to remove the solvent. The sample was then diluted with DI water and its concentration was measured by a TOC analyzer. The methyl palmitate concentration was detected by a UV–Vis spectrophotometer (Libra S32, Biochrom Ltd., England) at the wavelength of 232 nm where methyl palmitate has the strongest absorbance. Calibration curves were attained for all solutes in their respective solvents prior to the concentration determination. This enabled us precisely calculating the solute concentration in each feed solution.

To evaluate the feasibility of using OSFO to concentrate pharmaceuticals and recover organic solvents, tetracycline and triglycerides dissolved in alcohols and hexane, respectively, were chosen as the model feed solutions. In the ethanol system, 2 M LiCl was utilized as the draw solution and tetracycline dissolved in ethanol was adopted as the feed solution. The tetracycline concentration was varied from 1000, 2000, 5000 to 10000 ppm. On the other hand, 2 M LiCl and 2000 ppm tetracycline in IPA were utilized as the draw and feed solutions, respectively, in the IPA system. As in the hexane system, 50% methyl palmitate and 20% triglycerides solution were adopted as feeds.

The experimental tests were conducted under the FO mode. The solute rejection *R* (%) was employed to quantify the membrane rejection to the feed solute (i.e., tetracycline or triglycerides) and it was defined as the percentage of the feed solute that was retained by the membrane as follows:

3 $$R = 1 - \frac{{C_{\mathrm d} \times V_{\mathrm d}/V_{\mathrm p}}}{{C_{\mathrm f}}}$$

where *C*_d_ is the feed solute (i.e., tetracycline or triglycerides) concentration in the draw solution at the end of each OSFO test, *V*_d_ is the final volume of the draw solution, *V*_p_ is the volume of the permeate, and *C*_f_ is the tetracycline or triglycerides concentration in the feed solution. *C*_d_ of tetracycline was determined with the aid of a UV–Vis spectrophotometer at the wavelength of 366 nm where tetracycline has the strongest absorbance. *C*_d_ of triglycerides was measured with the aid of a HPLC-VWD at the wavelength of 208 nm. In addition, measures such as elongating the testing duration or reducing the feed volume had been taken in this study to ensure data reliability.

### Determination of the osmotic pressure

The osmotic pressures of draw solutions were determined by a lab-built direct membrane osmometer^39^. During the measurements, the draw solution was injected slowly into the fluid chamber until it was fully filled. A lab-fabricated TFC membrane was then utilized to cover the chamber in such a way that the selective layer was in direct contact with the draw solution. Subsequently, the membrane was kept in place with a stainless steel wire mesh. The assembled osmometer was then placed in the solvent of the draw solution and connected with a pressure transducer (Omega, USA). The pressure changes in the chamber were detected and recorded by the transducer. After reaching the equilibrium, the final pressure of the chamber was recorded as the osmotic pressure of the draw solution.

### Determination of transport properties in the membrane

Pure solvent permeance*, A*(L m^−2^ h^−1^ bar^−1^, LMH bar^−1^), of the TFC membranes were determined under a pressure-driven process by testing the membranes under a trans-membrane pressure, Δ*P*, of 10.0 bar in dead-end cells at room temperature. It was assumed that the substrate posed little transport resistance and the permeability/permeance was mainly determined by the selective layer. The pure solvent permeance, *A*, was calculated as per Eq. (4):

4 $$A = \frac{{\Delta V}}{{\Delta t}}\frac{1}{{A_{\mathrm m}\Delta P}},$$

where Δ*V* (g) is the volume of permeated solvent, Δ*t* (h) is the test duration, *A*_m_ is the effective area of the testing cell, and Δ*P* (bar) is the applied trans-membrane pressure.

As solvent permeability is mainly governed by the polyamide selective layer, the solvent solubility and diffusivity in the selective layer are determined with the aid of polyamide layers fabricated from free-standing interfacial polymerization. The fabrication procedure of the polyamide layer can be found in the Supplementary Methods^47^. For the solvent solubility tests, the polyamide layers were weighed immediately after drying in a vacuum oven to avoid moisture absorption (*m*_d_). The films were then immersed in excessive respective organic solvents at room temperature for at least one week to be fully saturated with the organic solvents. Two different methods were employed to determine the solvent solubility in the selective layer. For the first method, the organic solvent was vaporized at room temperature and the weight change was recorded. As the evaporation rate would change at different interfaces, the weight profile would display three different weight loss rates. The first two slopes represented the solvent evaporation above the membrane surface and from the inside of the membrane, respectively. The third slope was flat, indicating the organic solvent was fully evaporated, and the weight was recorded as *m*_d_’. Subsequently, the weight at the interception point of the first two slopes (i.e., the film surface) was calculated as the weight of the wet film (*m*_w_). As an alternative method, this procedure was repeated except that the organic solvent was poured away after the membrane was fully saturated and the film was wiped dry with a filter paper. The data were only considered as valid when the difference between these two methods was lower than 0.1g. Each measurement was conducted on at least five different polyamide films fabricated from separate free-standing interfacial polymerization. The solubility of the solvent in a membrane (*S*, gram solvent per gram membrane, g g^−1^) was calculated based on the weights of wet and dry films:

5 $${{S}} = \frac{{m_{\mathrm w} - m_{\mathrm d}^\prime }}{{m_{\mathrm d}}}$$

The organic solvent diffusivity (*D*_s_) was determined based on the solution-diffusion model^48–50^ as follows:

6 $$D_{\mathrm s} = \frac{{A \cdot \Delta x \cdot RT}}{{C_{\mathrm s} \cdot \overline {V_{\mathrm s}} }},$$

where *A* (LMH bar^−1^) is the pure solvent permeance, Δ*x* (m) is the thickness of the selective layer of the TFC membrane, *C*_s_(g m^−3^) is the solvent concentration in the membrane, and $\overline {V_{\mathrm{s}}}$ (m^3^ mol^−1^) is the partial molar volume of the organic solvent. An example can be found in the Supplementary Note 1.

For all experiments, each measurement was conducted using at least three different membrane coupons, and the interfacial polymerization was conducted separately for each membrane coupon.

### Data availability

The authors declare that all data supporting the findings of this study are available within the paper and its supplementary information files.

## Electronic supplementary material

Supplementary Information
