# Supplementary material for: Pharmaceutical concentration using organic solvent forward osmosis for solvent recovery
Source: Nat Commun. 2018 Apr 12;9:1426. doi: 10.1038/s41467-018-03612-2 (PMC5897543; doi:10.1038/s41467-018-03612-2)
Supplement: Supplementary file 1 — Supplementary Information [file 41467_2018_3612_MOESM1_ESM.pdf]

Pharmaceutical Concentration using Organic Solvent Forward Osmosis for Solvent Recovery

Yue Cui, Tai-Shung Chung

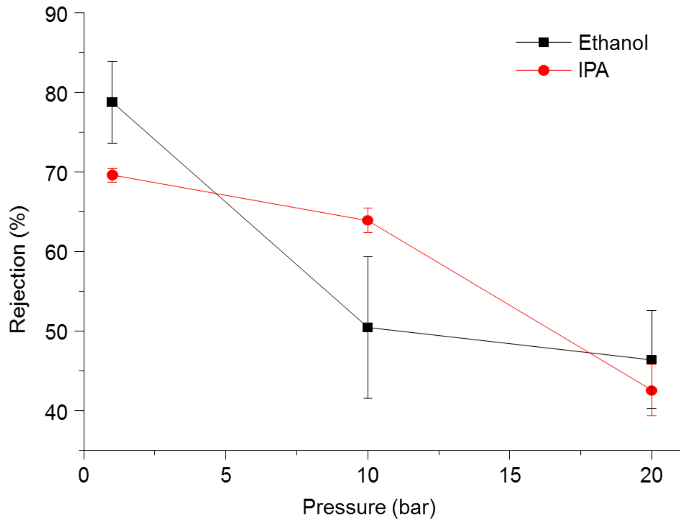

Supplementary Figure 1. Tetracycline rejection in the pressure driven process as a function of operating pressure. Tetracycline rejections *R (%)* of the TFC membranes were determined under a pressure driven process by testing the membranes under a trans-membrane pressure, ΔP, varying from 1-20 bar in dead-end cells at room temperature. In the tests, 50 ppm tetracycline in ethanol or IPA was used as the feed solution. The solute rejection R was calculated based on the equation: $R = \left(1 - \frac{C_p}{C_f}\right) \times 100\%$. The concentrations of tetracycline in the feed (C_f) and permeate (C_p) were determined by a UV–Vis spectrophotometer at the wavelength of 366 nm where tetracycline has the strongest absorbance. The rejection of tetracycline declines when applying a higher operating pressure, suggesting the pressure is another driving force in this process and thus, the pore flow mechanism plays a significant role.

Supplementary Table 1. Physical properties of the solvents [1, 2, 3]

Solvent	Dynamic size (Å)	Viscosity (mPa·s)	Solubility parameter ($(MPa)^{1/2}$)
Water	2.75	0.89	47.8
Ethanol	4.5	1.10	26.5
Isopropanol	4.7	2.04	23.8
Hexane	4.3	0.33	14.9

Supplementary Table 2. Rejections of commercial OSN membranes*

Membrane	Solvent flux (LMH)	Rejection (%)
DuraMem 300	1.98 ± 0.03	88.16 ± 2.03

*Feed solution: 50 ppm tetracycline in ethanol; pressure: 5 bar

Supplementary Table 3. Solutes transport properties in the membrane

Solvent	Solute	Flux (*B*, LMH)
Ethanol	Tetracycline	0.098±0.040
	LiCl [a]	0.066±0.023
	CA [b]	0.134±0.038
	PEG1000 [c]	0.416±0.092
IPA	Tetracycline	0.015±0.004
	LiCl	0.035±0.024
	DEA [d]	~0
Hexane	Methyl Palmitate	~0
	Soya Lecithin	~0

([a]LiCl: lithium chloride; [b]CA: citric acid; [c]PEG1000: polyethylene glycol 1000; [d]DEA: diethanolamine)

Supplementary Note 1

An example of solvent diffusivity calculation

The organic solvent diffusivity (D_s) was determined based on the solution-diffusion model as follows:

$$D_s = \frac{A \cdot \Delta x \cdot RT}{C_s \cdot \bar{V}_s} \quad (1)$$

where $A(LMH \cdot bar^{-1})$ is the pure solvent permeance, $\Delta x(m)$ is the thickness of the selective layer of the TFC membrane, $Cs(g \cdot m^{-3})$ is the solvent concentration in the membrane, and $\bar{V}_s$ $(m^3 \cdot mol^{-1})$ is the partial molar volume of the organic solvent.

For example, the permeability (38.76 $\times 10^{-9}$ LMH·bar^{-1}·m) of ethanol in the membrane was first obtained by multiplying the permeance (A, 0.23 LMH·bar^{-1}) with the thickness of the selective layer (Δx, 168.75 nm). Subsequently, the solvent concentration in the membrane Cs (4.16 $\times 10^7$ g·m^{-3}) was determined via the division of the amount of solvent absorbed by the polyamide film (g) by the polyamide volume (2.27 $\times 10^{-8}$ m^3). Next, gas constant (R=8.134 J·K^{-1}·mol^{-1}) and room temperature (298K) and the partial molar volume of ethanol (5.83$\times 10^{-5}$ m^3·mol^{-1}) were substituted into the Supplementary Equation 1. Finally, unit conversion was conducted and the ethanol diffusivity (8.68$\times 10^{-9}$ m^2·s^{-1}) was obtained.

Supplementary Methods

Free-standing Interfacial Polymerization of the Polyamide Films

The formation of the polyamide film was achieved by a free-standing interfacial polymerization reaction between the MPD aqueous and TMC hexane solutions. No substrate was utilized during the membrane formation. First, a 40 mL 2% MPD aqueous solution containing 0.1% SDS was poured into a petri dish and allowed to stabilize the liquid surface. Subsequently, a 40 mL 0.1% TMC hexane solution was added on the top surface of the MPD solution dropwise. The petri dish was then covered with a lid. The growth of the thin film was clearly observed at the interface. After a 24 h reaction, the petri dish was drained and only a thin film remained in. The resultant thin film was rinsed several times by ethanol to remove the excess monomers and dried in a vacuum oven for further usage.

Solute transport properties

Solute flux *(B, $L\cdot m^{-2}\cdot h^{-1}$, LMH)* can be determined in the OSFO process based on the solution diffusion model. In this part, all the testing solutes were adopted as the draw solutes and respective organic solvents as the feed solution. Subsequently, the solvent flux, J_w *(LMH)*, and reverse solute flux, J_s ($g\cdot m^{-2}\cdot h^{-1}$, *gMH*), were obtained. *B* was calculated based on the given Supplementary Equation (2)[4]:

$$B = \frac{J_s}{J_w} \cdot A \cdot iRT \qquad (2)$$

where *A*($LMH\cdot bar^{-1}$) is the pure solvent permeance, *i* is the van’t Hoff factor, which is assumed to be 1 in this study, *R* is the gas constant and *T(K)* is the room temperature.

Supplementary References:

1. Darvishmanesh, S., Degreve, J., Van der Bruggen, B. Mechanisms of solute rejection in solvent resistant nanofiltration: the effect of solvent on solute rejection. *Phys Chem Chem Phys* **12**, 13333-13342 (2010).

2. Liu, R., *et al.* Graphene oxide membrane for liquid phase organic molecular separation. *Carbon* **77**, 933-938 (2014).

3. Wu, H., Gong, Q., Olson, D. H., Li, J. Commensurate Adsorption of Hydrocarbons and Alcohols in Microporous Metal Organic Frameworks. *Chem. Rev.* **112**, 836-868 (2012).

4. Zhang, S., Fu, F., Chung, T. S. Substrate modifications and alcohol treatment on thin film composite membranes for osmotic power. *Chem. Eng. Sci.* **87**, 40-50 (2013).
